# 1-{6-Chloro-2-[(2-chloro-8-methyl-3-quinol­yl)meth­oxy]-4-phenyl­quinolin-3-yl}ethanone

**DOI:** 10.1107/S160053681002595X

**Published:** 2010-07-07

**Authors:** F. Nawaz Khan, Venkatesha R. Hathwar, Rajesh Kumar, A. Sudheer Kumar, Mehmet Akkurt

**Affiliations:** a Organic and Medicinal Chemistry Research Laboratory, Organic Chemistry Division, School of Advanced Sciences, VIT University, Vellore 632 014, Tamil Nadu, India; b Solid State and Structural Chemistry Unit, Indian Institute of Science, Bangalore 560 012, Karnataka, India; c Department of Physics, Faculty of Arts and Sciences, Erciyes University, 38039 Kayseri, Turkey

## Abstract

In the title mol­ecule, C_28_H_20_Cl_2_N_2_O_2_, the dihedral angle between the 2-chloro­quinoline and 6-chloro­quinoline rings is 7.55 (6)°. The dihedral angle between the phenyl ring and its attached quinoline ring is 62.59 (4)°. In the crystal, aromatic π–π stacking inter­actions [centroid–centroid distances = 3.771 (3) and 3.612 (3) Å] help to establish the packing.

## Related literature

For the structures of related 2-quinolone compounds, see: Khan, Roopan, Hathwar *et al.* (2010 ▶); Khan, Roopan, Kumar *et al.* (2010 ▶). For the biological activity, see: Ukita & Mizuno (1960 ▶); Jayashree *et al.* (2010 ▶); Joseph *et al.* (2002 ▶); Xiao *et al.* (2001 ▶). For related literature, see: Roopan & Khan (2009 ▶). For bond-length data, see: Allen *et al.* (1987 ▶).
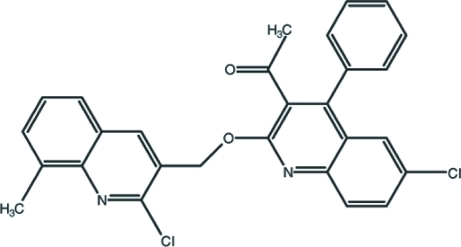


## Experimental

### 

#### Crystal data

C_28_H_20_Cl_2_N_2_O_2_
*M* *_r_* = 487.36
Triclinic, 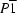

*a* = 9.7396 (4) Å
*b* = 10.5520 (3) Å
*c* = 13.0108 (4) Å
α = 88.730 (3)°
β = 68.127 (4)°
γ = 71.105 (4)°
*V* = 1166.62 (8) Å^3^
*Z* = 2
Mo *K*α radiation
μ = 0.31 mm^−1^
*T* = 295 K
0.24 × 0.18 × 0.15 mm

#### Data collection

Oxford Xcalibur Eos (Nova) CCD detector diffractometer
Absorption correction: multi-scan (*CrysAlis PRO RED*; Oxford Diffraction, 2009 ▶) *T*_min_ = 0.930, *T*_max_ = 0.955
22437 measured reflections
4323 independent reflections
2707 reflections with *I* > 2σ(*I*)
*R*_int_ = 0.043

#### Refinement

*R*[*F* ^2^ > 2σ(*F* ^2^)] = 0.040
*wR*(*F* ^2^) = 0.099
*S* = 0.95
4323 reflections
309 parameters
H-atom parameters constrained
Δρ_max_ = 0.17 e Å^−3^
Δρ_min_ = −0.20 e Å^−3^

### 

Data collection: *CrysAlis PRO CCD* (Oxford Diffraction, 2009 ▶); cell refinement: *CrysAlis PRO CCD*; data reduction: *CrysAlis PRO RED* (Oxford Diffraction, 2009 ▶); program(s) used to solve structure: *SHELXS97* (Sheldrick, 2008 ▶); program(s) used to refine structure: *SHELXL97* (Sheldrick, 2008 ▶); molecular graphics: *ORTEP-3* (Farrugia, 1997 ▶); software used to prepare material for publication: *WinGX* (Farrugia, 1999 ▶) and *PLATON* (Spek, 2009 ▶).

## Supplementary Material

Crystal structure: contains datablocks global, I. DOI: 10.1107/S160053681002595X/lx2158sup1.cif

Structure factors: contains datablocks I. DOI: 10.1107/S160053681002595X/lx2158Isup2.hkl

Additional supplementary materials:  crystallographic information; 3D view; checkCIF report

## Figures and Tables

**Fig. 1.**
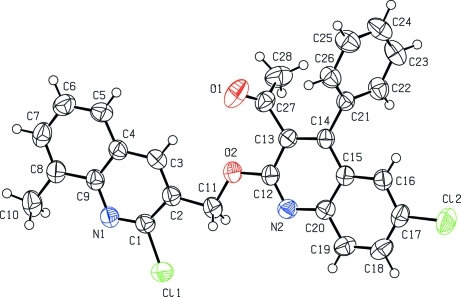
The molecular structure of the title compound with the atom numbering scheme. Displacement ellipsoids are drawn at the 50% probability level. H atoms are presented as a small spheres of arbitrary radius.

**Fig. 2.**
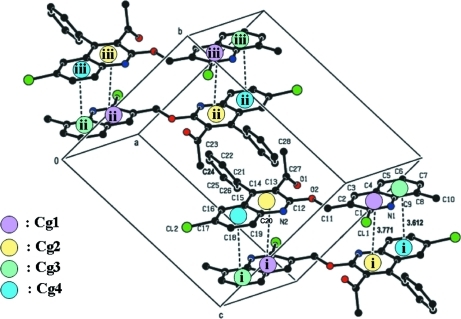
π–π interactions (dotted lines) in the crystal structure of the title compound. Cg denotes the ring centroids. [Symmetry codes: (i ) - x, - y + 1, - z + 2; (ii) - x + 1, - y + 1, - z + 1; (iii) x + 1, y, z - 1.]

## supplementary crystallographic information

### Comment

In continuation of our previous work (Roopan *et al.*, 2009; Khan, Roopan, Hathwar *et al.*, 2010; Khan, Roopan, Kumar *et al.*, 2010), we here report the molecular and crystal structure of 1-{6-chloro-2-[(2-chloro-8-methylquinolin-3-yl)methoxy]-4- phenylquinolin-3-yl}ethan-1-one. In the title molecule (I), (Fig. 1), the bond lengths (Allen *et al.*, 1987) and angles observed are within normal ranges and are consistent with those related structures (Roopan *et al.*, 2009; Khan, Roopan, Hathwar *et al.*, 2010; Khan, Roopan, Kumar *et al.*, 2010).

The quinoline rings (N1/C1-C9) and (N2/C12–C20) rings in (I) are almost planar, with maximal deviations from their mean planes of 0.016 (2) and of 0.042 (2) Å, respectively. These rings make a dihedral angle of 7.55 (6)° with each other. The N2/C12-C20 quinoline ring makes a dihedral angle of 62.59 (4)° with the C21-C26 phenyl ring. The molecular packing (Fig. 2) is stabilized by π–π stacking interactions between the quinoline rings of the adjacent molecules [Cg1···Cg2^i^ = 3.771 (3) Å and Cg3···Cg4^i^ = 3.612 (3) Å; where the Cg1, Cg2, Cg3 and Cg4 are the centroids of the N1/C1-C4/C9, N2/C12-C15/C20, C4-C9 and C15-C20 rings, respectively].

### Experimental

To a solution of 1-(6-chloro-2-hydroxy-4-phenylquinolin-3-yl)ethanone (1 mmol) in DMSO (5 ml) solution 2-chloro-3-chloromethyl-8-methylquinoline (1 mmol), Ag_2_SO_4_ (10 mol %) were added and refluxed at 383 K. The reaction was completed with in 20 min. The reaction mixture was then filtered and the supernatant liquid was added drop wise in to the crushed ice. The solution was neutralized with dilute HCl. The precipitate was filtered off and re-crystallized with ethanol. The clear solution was kept for a day and the resulting crystals were dried.

### Refinement

H atoms were positioned with idealized geometry using a riding model with C–H = 0.93-0.97 Å and refined as riding with *U_iso_*(H) = 1.2-1.5*U_eq_*(C).

### Crystal data

**Table:**

C_28_H_20_Cl_2_N_2_O_2_	*Z* = 2
*M**_r_* = 487.36	*F*(000) = 504
Triclinic, *P*1	*D*_x_ = 1.387 Mg m^−^^3^
Hall symbol: -P 1	Mo *K*α radiation, λ = 0.71073 Å
*a* = 9.7396 (4) Å	Cell parameters from 1523 reflections
*b* = 10.5520 (3) Å	θ = 1.9–21.4°
*c* = 13.0108 (4) Å	µ = 0.31 mm^−^^1^
α = 88.730 (3)°	*T* = 295 K
β = 68.127 (4)°	Block, colourless
γ = 71.105 (4)°	0.24 × 0.18 × 0.15 mm
*V* = 1166.62 (8) Å^3^	

### Data collection

**Table:**

Oxford Xcalibur Eos (Nova) CCD detector diffractometer	4323 independent reflections
Radiation source: Enhance (Mo) X-ray Source	2707 reflections with *I* > 2σ(*I*)
graphite	*R*_int_ = 0.043
ω scans	θ_max_ = 25.5°, θ_min_ = 3.3°
Absorption correction: multi-scan (*CrysAlis PRO* RED; Oxford Diffraction, 2009)	*h* = −11→11
*T*_min_ = 0.930, *T*_max_ = 0.955	*k* = −12→12
22437 measured reflections	*l* = −15→15

### Refinement

**Table:**

Refinement on *F*^2^	Primary atom site location: structure-invariant direct methods
Least-squares matrix: full	Secondary atom site location: difference Fourier map
*R*[*F*^2^ > 2σ(*F*^2^)] = 0.040	Hydrogen site location: difference Fourier map
*wR*(*F*^2^) = 0.099	H-atom parameters constrained
*S* = 0.95	*w* = 1/[σ^2^(*F*_o_^2^) + (0.0508*P*)^2^] where *P* = (*F*_o_^2^ + 2*F*_c_^2^)/3
4323 reflections	(Δ/σ)_max_ < 0.001
309 parameters	Δρ_max_ = 0.17 e Å^−^^3^
0 restraints	Δρ_min_ = −0.20 e Å^−^^3^

### Special details

**Table:**

Geometry. Bond distances, angles *etc*. have been calculated using the rounded fractional coordinates. All su's are estimated from the variances of the (full) variance-covariance matrix. The cell e.s.d.'s are taken into account in the estimation of distances, angles and torsion angles
Refinement. Refinement on *F*^2^ for ALL reflections except those flagged by the user for potential systematic errors. Weighted *R*-factors *wR* and all goodnesses of fit *S* are based on *F*^2^, conventional *R*-factors *R* are based on *F*, with *F* set to zero for negative *F*^2^. The observed criterion of *F*^2^ > σ(*F*^2^) is used only for calculating -*R*-factor-obs *etc*. and is not relevant to the choice of reflections for refinement. *R*-factors based on *F*^2^ are statistically about twice as large as those based on *F*, and *R*-factors based on ALL data will be even larger.

### Fractional atomic coordinates and isotropic or equivalent isotropic displacement parameters (Å^2^)

**Table:**

	*x*	*y*	*z*	*U*_iso_*/*U*_eq_	
Cl1	0.16697 (6)	0.85124 (6)	1.16788 (4)	0.0619 (2)	
Cl2	0.24300 (8)	0.10446 (6)	0.58993 (5)	0.0766 (3)	
O1	0.8426 (2)	0.46569 (18)	0.71113 (15)	0.0882 (8)	
O2	0.51617 (15)	0.59876 (14)	0.85708 (11)	0.0569 (5)	
N1	0.40686 (19)	0.91617 (16)	1.15591 (12)	0.0469 (6)	
N2	0.34893 (19)	0.50251 (15)	0.83264 (13)	0.0467 (6)	
C1	0.3644 (2)	0.83966 (19)	1.10679 (15)	0.0442 (7)	
C2	0.4616 (2)	0.74620 (18)	1.01018 (15)	0.0439 (7)	
C3	0.6156 (2)	0.73538 (19)	0.96840 (15)	0.0478 (7)	
C4	0.6722 (2)	0.81378 (19)	1.01774 (15)	0.0453 (7)	
C5	0.8312 (2)	0.8047 (2)	0.97723 (17)	0.0556 (8)	
C6	0.8776 (3)	0.8850 (2)	1.02774 (19)	0.0640 (9)	
C7	0.7671 (3)	0.9777 (2)	1.11959 (18)	0.0610 (9)	
C8	0.6118 (3)	0.9910 (2)	1.16341 (16)	0.0514 (8)	
C9	0.5621 (2)	0.90695 (19)	1.11163 (15)	0.0441 (7)	
C10	0.4943 (3)	1.0894 (2)	1.26265 (18)	0.0675 (9)	
C11	0.3932 (2)	0.6686 (2)	0.96029 (15)	0.0522 (7)	
C12	0.4862 (2)	0.51534 (18)	0.79707 (16)	0.0442 (7)	
C13	0.6141 (2)	0.45183 (18)	0.69549 (15)	0.0421 (7)	
C14	0.5961 (2)	0.36140 (18)	0.63171 (15)	0.0404 (6)	
C15	0.4501 (2)	0.33709 (18)	0.67024 (15)	0.0413 (7)	
C16	0.4214 (2)	0.2410 (2)	0.61547 (16)	0.0477 (7)	
C17	0.2772 (3)	0.2266 (2)	0.65504 (17)	0.0514 (8)	
C18	0.1544 (3)	0.3075 (2)	0.74891 (18)	0.0577 (8)	
C19	0.1802 (2)	0.3982 (2)	0.80495 (17)	0.0529 (8)	
C20	0.3284 (2)	0.41356 (19)	0.76929 (16)	0.0430 (7)	
C21	0.7258 (2)	0.29310 (18)	0.52394 (15)	0.0411 (7)	
C22	0.7071 (2)	0.3150 (2)	0.42376 (17)	0.0540 (8)	
C23	0.8307 (3)	0.2580 (2)	0.32397 (17)	0.0645 (9)	
C24	0.9741 (3)	0.1765 (2)	0.32318 (18)	0.0651 (9)	
C25	0.9930 (3)	0.1522 (2)	0.42123 (18)	0.0635 (8)	
C26	0.8697 (2)	0.2107 (2)	0.52146 (17)	0.0536 (8)	
C27	0.7577 (2)	0.4940 (2)	0.66076 (18)	0.0524 (7)	
C28	0.7825 (3)	0.5774 (2)	0.56593 (19)	0.0723 (9)	
H3	0.68530	0.67470	0.90570	0.0570*	
H5	0.90470	0.74350	0.91580	0.0670*	
H6	0.98280	0.87830	1.00120	0.0770*	
H7	0.80110	1.03260	1.15220	0.0730*	
H10A	0.54610	1.13850	1.28720	0.1010*	
H10B	0.44770	1.04160	1.32170	0.1010*	
H10C	0.41380	1.15120	1.24260	0.1010*	
H11A	0.35880	0.60500	1.00970	0.0630*	
H11B	0.30370	0.72910	0.94720	0.0630*	
H16	0.50110	0.18710	0.55200	0.0570*	
H18	0.05540	0.29910	0.77290	0.0690*	
H19	0.09850	0.45120	0.86800	0.0630*	
H22	0.61010	0.36870	0.42390	0.0650*	
H23	0.81750	0.27460	0.25720	0.0770*	
H24	1.05770	0.13810	0.25590	0.0780*	
H25	1.08920	0.09610	0.42070	0.0760*	
H26	0.88410	0.19420	0.58790	0.0640*	
H28A	0.86650	0.61000	0.55890	0.1090*	
H28B	0.68770	0.65240	0.57950	0.1090*	
H28C	0.80920	0.52340	0.49830	0.1090*	

### Atomic displacement parameters (Å^2^)

**Table:**

	*U*^11^	*U*^22^	*U*^33^	*U*^12^	*U*^13^	*U*^23^
Cl1	0.0520 (3)	0.0709 (4)	0.0580 (4)	−0.0276 (3)	−0.0098 (3)	−0.0047 (3)
Cl2	0.0897 (5)	0.0851 (5)	0.0828 (5)	−0.0534 (4)	−0.0430 (4)	0.0057 (3)
O1	0.0757 (12)	0.1028 (14)	0.1176 (15)	−0.0422 (10)	−0.0617 (12)	0.0225 (11)
O2	0.0572 (9)	0.0598 (9)	0.0529 (9)	−0.0249 (7)	−0.0152 (7)	−0.0134 (7)
N1	0.0549 (11)	0.0459 (10)	0.0428 (10)	−0.0199 (8)	−0.0197 (8)	0.0048 (8)
N2	0.0471 (10)	0.0458 (10)	0.0478 (10)	−0.0193 (8)	−0.0161 (8)	0.0052 (8)
C1	0.0488 (12)	0.0439 (11)	0.0408 (12)	−0.0193 (10)	−0.0156 (10)	0.0086 (10)
C2	0.0510 (13)	0.0422 (11)	0.0408 (11)	−0.0184 (10)	−0.0181 (10)	0.0062 (9)
C3	0.0511 (13)	0.0486 (12)	0.0386 (11)	−0.0150 (10)	−0.0134 (10)	0.0014 (9)
C4	0.0524 (13)	0.0504 (12)	0.0379 (11)	−0.0206 (10)	−0.0202 (10)	0.0116 (9)
C5	0.0545 (14)	0.0657 (15)	0.0462 (13)	−0.0266 (12)	−0.0140 (11)	0.0126 (11)
C6	0.0617 (15)	0.0831 (18)	0.0611 (15)	−0.0396 (14)	−0.0269 (13)	0.0222 (13)
C7	0.0809 (17)	0.0671 (15)	0.0625 (15)	−0.0458 (14)	−0.0407 (14)	0.0208 (12)
C8	0.0672 (15)	0.0516 (13)	0.0487 (13)	−0.0302 (12)	−0.0286 (11)	0.0145 (10)
C9	0.0558 (13)	0.0446 (12)	0.0395 (11)	−0.0217 (10)	−0.0229 (10)	0.0114 (9)
C10	0.0873 (17)	0.0623 (15)	0.0627 (15)	−0.0309 (13)	−0.0346 (13)	−0.0014 (12)
C11	0.0544 (13)	0.0519 (12)	0.0483 (13)	−0.0168 (11)	−0.0183 (11)	−0.0042 (10)
C12	0.0500 (13)	0.0403 (11)	0.0458 (12)	−0.0172 (10)	−0.0207 (10)	0.0025 (9)
C13	0.0418 (11)	0.0413 (11)	0.0450 (12)	−0.0144 (9)	−0.0185 (9)	0.0062 (9)
C14	0.0448 (11)	0.0386 (11)	0.0411 (11)	−0.0144 (9)	−0.0201 (9)	0.0060 (9)
C15	0.0486 (12)	0.0386 (11)	0.0418 (11)	−0.0174 (9)	−0.0212 (10)	0.0098 (9)
C16	0.0550 (13)	0.0499 (12)	0.0447 (12)	−0.0227 (11)	−0.0222 (10)	0.0075 (9)
C17	0.0618 (14)	0.0523 (13)	0.0585 (14)	−0.0310 (12)	−0.0337 (12)	0.0147 (11)
C18	0.0505 (13)	0.0629 (15)	0.0703 (16)	−0.0301 (12)	−0.0261 (12)	0.0192 (12)
C19	0.0463 (13)	0.0521 (13)	0.0592 (14)	−0.0206 (11)	−0.0161 (11)	0.0068 (10)
C20	0.0474 (12)	0.0401 (11)	0.0464 (12)	−0.0196 (10)	−0.0197 (10)	0.0118 (9)
C21	0.0455 (12)	0.0387 (11)	0.0418 (12)	−0.0178 (9)	−0.0168 (10)	0.0040 (9)
C22	0.0565 (13)	0.0517 (13)	0.0523 (14)	−0.0123 (11)	−0.0245 (11)	0.0033 (10)
C23	0.0849 (18)	0.0627 (15)	0.0420 (13)	−0.0204 (14)	−0.0241 (13)	0.0041 (11)
C24	0.0752 (17)	0.0533 (14)	0.0475 (14)	−0.0153 (13)	−0.0081 (12)	−0.0024 (11)
C25	0.0551 (14)	0.0559 (14)	0.0611 (16)	−0.0030 (11)	−0.0159 (12)	0.0009 (12)
C26	0.0556 (14)	0.0545 (13)	0.0464 (13)	−0.0124 (11)	−0.0205 (11)	0.0053 (10)
C27	0.0436 (12)	0.0462 (12)	0.0654 (14)	−0.0130 (10)	−0.0201 (11)	−0.0070 (10)
C28	0.0627 (15)	0.0676 (15)	0.0818 (17)	−0.0323 (13)	−0.0143 (13)	0.0140 (13)

### Geometric parameters (Å, °)

**Table:**

Cl1—C1	1.749 (2)	C18—C19	1.354 (3)
Cl2—C17	1.740 (3)	C19—C20	1.405 (3)
O1—C27	1.199 (3)	C21—C22	1.386 (3)
O2—C11	1.433 (2)	C21—C26	1.378 (3)
O2—C12	1.355 (2)	C22—C23	1.378 (3)
N1—C1	1.291 (3)	C23—C24	1.379 (4)
N1—C9	1.373 (3)	C24—C25	1.363 (3)
N2—C12	1.294 (3)	C25—C26	1.383 (3)
N2—C20	1.371 (3)	C27—C28	1.491 (3)
C1—C2	1.419 (3)	C3—H3	0.9300
C2—C3	1.357 (3)	C5—H5	0.9300
C2—C11	1.495 (3)	C6—H6	0.9300
C3—C4	1.408 (3)	C7—H7	0.9300
C4—C5	1.408 (3)	C10—H10A	0.9600
C4—C9	1.415 (3)	C10—H10B	0.9600
C5—C6	1.359 (3)	C10—H10C	0.9600
C6—C7	1.398 (3)	C11—H11A	0.9700
C7—C8	1.362 (4)	C11—H11B	0.9700
C8—C9	1.422 (3)	C16—H16	0.9300
C8—C10	1.498 (3)	C18—H18	0.9300
C12—C13	1.421 (3)	C19—H19	0.9300
C13—C14	1.372 (3)	C22—H22	0.9300
C13—C27	1.511 (3)	C23—H23	0.9300
C14—C15	1.428 (3)	C24—H24	0.9300
C14—C21	1.489 (3)	C25—H25	0.9300
C15—C16	1.406 (3)	C26—H26	0.9300
C15—C20	1.416 (3)	C28—H28A	0.9600
C16—C17	1.363 (4)	C28—H28B	0.9600
C17—C18	1.396 (3)	C28—H28C	0.9600
			
Cl1···C24^i^	3.563 (2)	C13···H26	3.0100
Cl1···H11A	2.9500	C15···H22	3.0600
Cl1···H11B	2.8100	C16···H10B^iii^	3.0600
Cl1···H24^i^	2.9600	C16···H22	3.0700
Cl1···H18^ii^	2.9500	C21···H16	2.6700
Cl1···H26^iii^	3.0800	C21···H28C	2.7800
Cl2···H25^iv^	3.1100	C22···H28C	3.0000
O1···O2	2.901 (2)	C22···H16	2.7900
O1···C18^v^	3.169 (4)	C24···H5^ix^	3.0900
O2···O1	2.901 (2)	C27···H26	3.0300
O1···H18^v^	2.6200	H3···O2	2.3300
O2···H3	2.3300	H3···H5	2.5200
N1···H10B	2.7700	H5···H3	2.5200
N1···H10C	2.7800	H5···C24^ix^	3.0900
N2···H11A	2.6100	H5···H24^ix^	2.5000
N2···H11B	2.6700	H7···H10A	2.3500
N2···H23^vi^	2.8900	H10A···H7	2.3500
C2···C8^vii^	3.598 (3)	H10B···N1	2.7700
C4···C19^iii^	3.543 (3)	H10B···C16^iii^	3.0600
C5···C19^iii^	3.504 (3)	H10C···N1	2.7800
C6···C18^iii^	3.484 (3)	H10C···C3^vii^	3.0400
C7···C18^iii^	3.439 (3)	H11A···Cl1	2.9500
C7···C17^iii^	3.579 (3)	H11A···N2	2.6100
C8···C2^vii^	3.598 (3)	H11B···Cl1	2.8100
C8···C17^iii^	3.475 (3)	H11B···N2	2.6700
C16···C22	3.275 (3)	H16···C21	2.6700
C17···C7^iii^	3.579 (3)	H16···C22	2.7900
C17···C8^iii^	3.475 (3)	H18···O1^iv^	2.6200
C18···O1^iv^	3.169 (4)	H18···Cl1^ii^	2.9500
C18···C6^iii^	3.484 (3)	H22···C15	3.0600
C18···C7^iii^	3.439 (3)	H22···C16	3.0700
C19···C5^iii^	3.504 (3)	H23···N2^vi^	2.8900
C19···C4^iii^	3.543 (3)	H24···Cl1^viii^	2.9600
C21···C28	3.313 (3)	H24···C5^ix^	2.9400
C22···C16	3.275 (3)	H24···H5^ix^	2.5000
C24···Cl1^viii^	3.563 (2)	H25···Cl2^v^	3.1100
C26···C27	3.173 (3)	H26···C13	3.0100
C27···C26	3.173 (3)	H26···C27	3.0300
C28···C21	3.313 (3)	H26···Cl1^iii^	3.0800
C3···H10C^vii^	3.0400	H28B···C10^vii^	3.0300
C5···H24^ix^	2.9400	H28C···C21	2.7800
C10···H28B^vii^	3.0300	H28C···C22	3.0000
			
C11—O2—C12	118.02 (17)	C22—C23—C24	120.0 (2)
C1—N1—C9	117.56 (16)	C23—C24—C25	119.9 (2)
C12—N2—C20	116.71 (17)	C24—C25—C26	120.3 (2)
Cl1—C1—N1	116.13 (15)	C21—C26—C25	120.7 (2)
Cl1—C1—C2	117.06 (16)	O1—C27—C13	120.6 (2)
N1—C1—C2	126.8 (2)	O1—C27—C28	122.5 (2)
C1—C2—C3	115.30 (18)	C13—C27—C28	116.8 (2)
C1—C2—C11	120.44 (18)	C2—C3—H3	119.00
C3—C2—C11	124.26 (17)	C4—C3—H3	119.00
C2—C3—C4	121.56 (17)	C4—C5—H5	120.00
C3—C4—C5	123.17 (18)	C6—C5—H5	120.00
C3—C4—C9	117.67 (19)	C5—C6—H6	120.00
C5—C4—C9	119.16 (19)	C7—C6—H6	120.00
C4—C5—C6	120.2 (2)	C6—C7—H7	119.00
C5—C6—C7	120.0 (3)	C8—C7—H7	119.00
C6—C7—C8	122.8 (2)	C8—C10—H10A	109.00
C7—C8—C9	117.67 (19)	C8—C10—H10B	109.00
C7—C8—C10	122.6 (2)	C8—C10—H10C	109.00
C9—C8—C10	119.8 (2)	H10A—C10—H10B	110.00
N1—C9—C4	121.05 (18)	H10A—C10—H10C	109.00
N1—C9—C8	118.75 (18)	H10B—C10—H10C	109.00
C4—C9—C8	120.2 (2)	O2—C11—H11A	110.00
O2—C11—C2	106.18 (17)	O2—C11—H11B	110.00
O2—C12—N2	119.90 (17)	C2—C11—H11A	111.00
O2—C12—C13	114.06 (18)	C2—C11—H11B	111.00
N2—C12—C13	126.01 (19)	H11A—C11—H11B	109.00
C12—C13—C14	117.91 (19)	C15—C16—H16	120.00
C12—C13—C27	118.15 (17)	C17—C16—H16	120.00
C14—C13—C27	123.84 (17)	C17—C18—H18	120.00
C13—C14—C15	118.45 (17)	C19—C18—H18	120.00
C13—C14—C21	120.10 (19)	C18—C19—H19	119.00
C15—C14—C21	121.44 (17)	C20—C19—H19	119.00
C14—C15—C16	123.51 (17)	C21—C22—H22	120.00
C14—C15—C20	117.99 (18)	C23—C22—H22	120.00
C16—C15—C20	118.50 (19)	C22—C23—H23	120.00
C15—C16—C17	120.23 (18)	C24—C23—H23	120.00
Cl2—C17—C16	120.35 (16)	C23—C24—H24	120.00
Cl2—C17—C18	118.4 (2)	C25—C24—H24	120.00
C16—C17—C18	121.3 (2)	C24—C25—H25	120.00
C17—C18—C19	119.7 (3)	C26—C25—H25	120.00
C18—C19—C20	121.1 (2)	C21—C26—H26	120.00
N2—C20—C15	122.68 (19)	C25—C26—H26	120.00
N2—C20—C19	118.18 (18)	C27—C28—H28A	110.00
C15—C20—C19	119.14 (19)	C27—C28—H28B	109.00
C14—C21—C22	121.01 (18)	C27—C28—H28C	109.00
C14—C21—C26	120.45 (17)	H28A—C28—H28B	109.00
C22—C21—C26	118.49 (18)	H28A—C28—H28C	109.00
C21—C22—C23	120.7 (2)	H28B—C28—H28C	109.00
			
C12—O2—C11—C2	177.13 (15)	N2—C12—C13—C27	171.72 (18)
C11—O2—C12—N2	1.1 (3)	C12—C13—C14—C15	0.5 (3)
C11—O2—C12—C13	179.27 (16)	C12—C13—C14—C21	179.35 (17)
C9—N1—C1—Cl1	178.38 (14)	C27—C13—C14—C15	−175.59 (18)
C9—N1—C1—C2	−0.4 (3)	C27—C13—C14—C21	3.2 (3)
C1—N1—C9—C4	−2.0 (3)	C12—C13—C27—O1	69.7 (3)
C1—N1—C9—C8	179.39 (18)	C12—C13—C27—C28	−107.3 (2)
C20—N2—C12—O2	−178.21 (16)	C14—C13—C27—O1	−114.2 (2)
C20—N2—C12—C13	3.9 (3)	C14—C13—C27—C28	68.9 (3)
C12—N2—C20—C15	0.8 (3)	C13—C14—C15—C16	−176.10 (18)
C12—N2—C20—C19	−179.37 (18)	C13—C14—C15—C20	3.5 (3)
Cl1—C1—C2—C3	−176.91 (14)	C21—C14—C15—C16	5.1 (3)
Cl1—C1—C2—C11	3.7 (2)	C21—C14—C15—C20	−175.27 (17)
N1—C1—C2—C3	1.9 (3)	C13—C14—C21—C22	−115.6 (2)
N1—C1—C2—C11	−177.46 (18)	C13—C14—C21—C26	61.7 (3)
C1—C2—C3—C4	−0.9 (3)	C15—C14—C21—C22	63.2 (3)
C11—C2—C3—C4	178.43 (18)	C15—C14—C21—C26	−119.5 (2)
C1—C2—C11—O2	172.29 (16)	C14—C15—C16—C17	−177.72 (19)
C3—C2—C11—O2	−7.0 (2)	C20—C15—C16—C17	2.7 (3)
C2—C3—C4—C5	179.57 (19)	C14—C15—C20—N2	−4.4 (3)
C2—C3—C4—C9	−1.3 (3)	C14—C15—C20—C19	175.73 (18)
C3—C4—C5—C6	178.91 (19)	C16—C15—C20—N2	175.24 (18)
C9—C4—C5—C6	−0.2 (3)	C16—C15—C20—C19	−4.6 (3)
C3—C4—C9—N1	2.8 (3)	C15—C16—C17—Cl2	−178.09 (15)
C3—C4—C9—C8	−178.59 (18)	C15—C16—C17—C18	1.1 (3)
C5—C4—C9—N1	−177.99 (18)	Cl2—C17—C18—C19	176.30 (17)
C5—C4—C9—C8	0.6 (3)	C16—C17—C18—C19	−2.9 (3)
C4—C5—C6—C7	−0.5 (3)	C17—C18—C19—C20	0.8 (3)
C5—C6—C7—C8	1.0 (3)	C18—C19—C20—N2	−176.95 (19)
C6—C7—C8—C9	−0.6 (3)	C18—C19—C20—C15	2.9 (3)
C6—C7—C8—C10	179.3 (2)	C14—C21—C22—C23	176.0 (2)
C7—C8—C9—N1	178.44 (18)	C26—C21—C22—C23	−1.4 (3)
C7—C8—C9—C4	−0.2 (3)	C14—C21—C26—C25	−176.8 (2)
C10—C8—C9—N1	−1.5 (3)	C22—C21—C26—C25	0.6 (3)
C10—C8—C9—C4	179.92 (19)	C21—C22—C23—C24	1.0 (3)
O2—C12—C13—C14	177.38 (17)	C22—C23—C24—C25	0.2 (4)
O2—C12—C13—C27	−6.3 (2)	C23—C24—C25—C26	−1.1 (4)
N2—C12—C13—C14	−4.6 (3)	C24—C25—C26—C21	0.7 (3)

Symmetry codes: (i) *x*−1, *y*+1, *z*+1; (ii) −*x*, −*y*+1, −*z*+2; (iii) −*x*+1, −*y*+1, −*z*+2; (iv) *x*−1, *y*, *z*; (v) *x*+1, *y*, *z*; (vi) −*x*+1, −*y*+1, −*z*+1; (vii) −*x*+1, −*y*+2, −*z*+2; (viii) *x*+1, *y*−1, *z*−1; (ix) −*x*+2, −*y*+1, −*z*+1.
